# Comments on the article: “Syrian refugees in Lebanon: the search for universal health coverage”

**DOI:** 10.1186/s13031-016-0090-9

**Published:** 2016-07-28

**Authors:** Walid Ammar, Alissar Radi, Fadi El-Jardali

**Affiliations:** 1 Ministry of Public Health, Beirut, Lebanon; 2 Faculty of Medical Sciences, Lebanese University, Beirut, Lebanon; 3 WHO, Beirut, Lebanon; 4 Faculty of Health Sciences, American University of Beirut, Beirut, Lebanon

**Keywords:** Fragmentation, Refugees, Lebanon

## Abstract

This letter intends to clarify information and misconceptions found in the article “Syrian refugees in Lebanon: the search for universal health coverage” which was published June 1st, 2016, and to challenge the core notion of fragmentation as presented by the authors. It also highlights the fact that the article does not recognize the severe shortage in refugees health financing and unmet promises by the international community, and calls for immediate action and far greater support from that community to address the needs of refugees in Lebanon.

Dear Editor,

On June 1^st^ 2016, Conflict and Health published an article entitled “Syrian refugees in Lebanon: the search for universal health coverage”.

The paper identifies the fragmentation of the health system in Lebanon as a main cause for not meeting the health needs of the diverse groups of population currently living in Lebanon (highlighted in Table 1 of that paper); and proposes “structural alternatives” as a solution for integrating the refugees in the health system in Lebanon (highlighted in Table 2 of that same paper).

While the authors of this paper highlight the strain that the health system in Lebanon faces by the rapid increase of its population by 30 % as a result of the massive influx of refugees, it is for this same reason that we believe that such structural reform is currently not at all feasible in Lebanon amidst political and social turmoil.

Additionally, we would like to clarify incorrect information and redress misconceptions found in this article, based on the same references cited by the authors. We would like to challenge the core notion presented in this paper that “in this segmented model of health system, the choice of provider and patient pathway is actually not determined by patient’s choice but by the social group classification”.

First, the Ministry of Public Health in Lebanon (MOPH), as stated in this paper, has contracts with both public and private providers, the same applies for the National Social Security Fund (NSSF). Consequently patients, of all social groups, have access to public and private providers and have the freedom to choose physicians and hospitals. As a result, patients covered by MOPH admitted to private hospitals represent 67 % of total admissions and this share is even higher among NSSF patients. Freedom of choice is also available to segments of the population with private insurance, which also has contacts with several autonomous public hospitals in addition to private hospitals.

Second, although the MOPH focuses on the vulnerable population, all Lebanese with no formal coverage are entitled to the ministry’s coverage, irrespective of their income. As a matter of fact people of all income levels seek MOPH services particularly for gaining access to expensive drugs that are provided by the MOPH for free to the uninsured.

These arguments undermine the main rationale behind the fragmentation of the health system by social groups as conceived by the authors in Table 1, and therefore question the relevance to the Lebanese context of the proposed solution adapted from Londono and Frenk (Table 2).

Finally, while the paper points out that the current humanitarian system [led by UNHCR] has also contributed to increase the fragmentation of the Lebanese health system, it is striking that the paper does not recognize the severe shortage in financing and unmet promises by the international community. Immediate action and far greater support from the international community is needed to address the needs of refugees in Lebanon.

